# In Memoriam: Jocelyn Anne Rankin (1946–2010) 

**DOI:** 10.3201/eid1612.IM1612

**Published:** 2010-12

**Authors:** Tanja Popovic

**Affiliations:** Author affiliation: Centers for Disease Control and Prevention, Atlanta, GA, USA

**Keywords:** Jocelyn Rankin, obituary, in memoriam

Jocelyn Anne Rankin, PhD, (figure) chief of the Information Center at the Centers for Disease Control and Prevention (CDC), died on September 19, 2010, at age 63 in her Florida home. Similar to the health professionals in the organization Médecins Sans Frontières, Jocelyn was a humanitarian and a librarian sans frontières. Although some make a difference by what they do, others, like Jocelyn, also make a difference by how they do it. She was a true leader and a mentor to many librarians (and not only to librarians but also to scientists and public health professionals) throughout the nation. These colleagues will remain profoundly grateful for her contributions to the world of information science, and will, just as deeply, cherish memories of how she made her professional mark—quietly, respectfully, and selflessly, yet with clear vision, determination, and passion. 

Born in Raleigh, North Carolina, USA, Dr Rankin achieved an education without borders as well: high school in Germany, a diploma in liberal arts from the American College in Paris, a BA cum laude in English from Hollins College in Virginia, an MLn in librarianship from Emory University in Atlanta, and a PhD in educational leadership from Georgia State University in Atlanta. Dr Rankin's career began at the Medical College of Georgia, Augusta, and continued at Georgetown University Medical Center, Washington, DC, and at Mercer University School of Medicine, Macon, Georgia. She designed and led the Georgia Interactive Network for Medical Information, the oldest statewide network of its kind in the nation. At CDC, she led in the creation of a state-of-the-art information center; guided implementation of an integrated, electronic information delivery system; helped create the US Department of Health and Human Services Library Consortium; and most recently, helped build the foundation for CDC’s Science Clips, a weekly digest of selected news pertinent to the public health community. Dr Rankin’s contribution as book review editor for Emerging Infectious Diseases was also highly valued.

She leaves behind many who feel privileged to have known her. We offer our condolences to her husband, William Rankin; daughters, Stephanie Smith and Kimberley Macdonald; son, William Rankin III; brother, Howell Cobb; and 2 grandchildren. 

**Figure:**
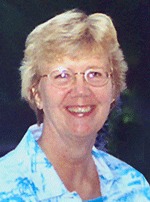
Jocelyn Anne Rankin

